# Interfacial Phenomena of Cotton/Polyester Blended Fabric Modified with Enzyme and Chitosan

**DOI:** 10.3390/polym18070867

**Published:** 2026-04-01

**Authors:** Anita Tarbuk, Ana Marija Grancarić, Stefana Begović, Tihana Dekanić

**Affiliations:** Department of Textile Chemistry and Ecology, University of Zagreb Faculty of Textile Technology, Prilaz baruna Filipovića 28a, HR-10000 Zagreb, Croatia; amgranca@ttf.hr (A.M.G.); stefana.begovic@ttf.unizg.hr (S.B.); tihana.dekanic@ttf.unizg.hr (T.D.)

**Keywords:** cotton/polyester blended fabric, pectinase, esterase, zeta potential, isoelectric point, specific surface charge, contact angle, wetting time, antimicrobial efficacy

## Abstract

In this study, the interfacial phenomena of cotton/polyester blended fabric modified with enzymes and chitosan were investigated. Enzymatic pretreatments (bioactivation) were carried out using a pectinase complex (Biosol PRO), an esterase complex (Texazym PES), and a combination of both. Bioactivation aimed to activate the surface and improve interfacial properties, primarily the hydrophilicity of the polyester component in the blend. For the functionalization of bio-activated blended fabrics, a homogenized chitosan solution in a 3% acetic acid was prepared and applied in a pad–dry–cure process. Changes after enzyme bioactivation, chitosan functionalization, and three washing cycles were monitored by interfacial phenomena—including zeta potential, isoelectric point (IEP), specific surface charge, and contact angle, as well as wetting time and maximum wetted radius—measured using a Moisture Management Tester (MMT). Mechanical and spectral properties of fabrics and antimicrobial efficacy were determined as well. Although esterase and pectinase act on different components of the fabric, both contribute to improved fabric properties, especially when used together. The presence of chitosan on the fabric after three washing cycles was confirmed on enzyme-bioactivated fabrics by zeta potential, IEP, and specific surface charge. The antimicrobial activity was confirmed as well. The best results were obtained after functionalization with chitosan on the esterase-bioactivated surface. Overall, these treatments provide flexible and mechanically stable functionalization, demonstrating both antimicrobial effectiveness and washing stability, with the possibility of easy implementation in the textile industry.

## 1. Introduction

Cotton and polyester blends account for a large share of the textile and clothing industry because they combine natural comfort with mechanical strength. However, the high crystallinity of polyester fibers results in few free active groups and hydrophobic properties, making surface modification necessary. The demand for highly efficient and environmentally sustainable textile materials has driven significant research into developing alternative and sustainable fiber surface modifications, particularly for polyester. Conventional chemical processing methods, such as alkaline treatment (e.g., cotton scouring and polyester hydrolysis), are effective but require high energy and chemical consumption, cause structural damage to fibers, and are environmentally unfriendly. Consequently, research has increasingly focused on developing equally effective but more environmentally sustainable alternatives, such as enzymes [1,2,3,4,5,6,7].

Enzymes can replace many hazardous and environmentally unfriendly chemicals in textile industry processes and, in some cases, reduce the need for high temperatures and pressures because they catalyze reactions at room temperature. The main difference between enzymes and chemical catalysts is that enzymes are sensitive to temperature and pH and have relatively low activation energy [2,3,4,5,6,7,8]. They are most often used at temperatures from 40 °C to 60 °C, which significantly reduces energy consumption compared to conventional methods. Additionally, they are biodegradable and do not pose any threat to the environment, making them energy, economically, and ecologically efficient (3E). For an enzyme to act on a specific substrate group, it must fit and bind to the substrate through Van der Waals forces and hydrogen bonds, forming an enzyme–substrate complex [7]. This complex is then broken down, the characteristic polymer bond is cleaved, and the enzyme remains unchanged after the reaction, continuing its activity until inhibited [2,7]. Although an enzyme acts on a specific site in the substrate, different types of enzymes can catalyze the same substrate if they have the appropriate active site structure. Depending on the type of chemical reaction they catalyze, enzymes are classified into six main groups: oxidoreductases (EC 1), transferases (EC 2), hydrolases (EC 3), lyases (EC 4), isomerases (EC 5), and ligases (EC 6). In textile pretreatment and modification, hydrolases are most commonly used because they catalyze the hydrolysis of various compounds. Common hydrolases include amylase, esterases, proteases, glycosidases, and lipases [6,7,8,9,10,11]. Another important group is lyase enzymes, specifically pectate lyase, which is crucial for eco-friendly bioscouring of cotton, replacing harsh alkali and caustic soda. These enzymes break down pectin, a glue-like substance on cotton fibers, to improve absorbency, whiteness, and dyeability. They function under neutral or mildly alkaline conditions (pH 8) and at lower temperatures, saving energy and reducing wastewater pollution [11,12,13,14,15,16,17,18,19,20,21].

In alkaline scouring of cotton, the bath contains NaOH (or Na_2_CO_3_), surfactants, and sequestrants. Surfactants lower the surface tension of water, enabling better wetting of the cotton fabric and helping to remove waxes by emulsifying them, while sequestrants bind polyvalent cations such as calcium, magnesium, iron, and other ions from their salts. The alkali removes part of the cuticle of the cotton fiber, with the possibility of damaging the cotton fiber by forming oxycellulose under alkaline conditions. Large amounts of water and energy are required, and alkaline wastewater requires special treatment procedures [13,14,15,16,17,18,19]. Enzymatic scouring (bio-scouring) produces more environmentally friendly wastewater, saves energy, and is compatible with other processes [19,20,21]. In the bio-scouring process, pectinase (pectate lyase) enzymes penetrate the micropores of the cuticle to reach the pectin, hydrolyzing it into water-soluble components. Surfactants and sequestrants then function as they do in alkaline scouring. The enzyme action specifically targets the pectin, and some waxes remaining on the surface give the cotton a soft touch, eliminating the need for an additional softening process [15,19,20,21]. Some studies reported good activity of pectinase and cutinase in degrading impurities in the cotton cuticle [22,23,24,25].

Surface modification of polyester fibers is usually performed by grafting, plasma treatment, and chemical reactions, including hydrolysis (alkaline or enzymatic) and aminolysis, to reduce hydrophobicity [7,26,27,28,29,30,31,32,33,34]. Hydrolysis cleaves ester bonds, resulting in significant physical and chemical changes, such as the formation of hydroxyl and carboxyl active groups on the surface of polyester fibers. Alkaline hydrolysis is the most common chemical process for modifying the surface of polyester fibers through a controlled degradation mechanism. This process is typically carried out in a 4–20% KOH or NaOH solution at high temperatures (130–140 °C) for 1–2 h. The mechanism relies on the nucleophilic action of hydroxyl ions (OH^−^) from alkali on the carbonyl carbon atoms in the polyester chain, resulting in the cleavage of ester bonds [27,28,29,30,31,32]. Chain cleavage causes physical and chemical changes, including the formation of microscopic cracks along the fiber and an increased number of available active groups, which significantly improve wetting and dyeing, as well as the touch and luster of the treated fabrics [7,28,32]. High temperature, the use of alkalis, and the long duration of the process are the main causes of the negative environmental impact, making the alkaline hydrolysis process environmentally unfavorable. Research indicates the potential use of cationic surfactants to accelerate the reaction and further reduce temperature and processing time, which would lessen the negative environmental impact [7,27,28,29,30,31,32]. However, enzymatic hydrolysis offers a biological alternative to alkaline hydrolysis by acting on a precisely defined polymer substrate and gradually degrading the polyester surface [7,8]. Hydrolases such as esterases, lipases, and cutinases can effectively hydrolyze ester bonds on the surface of polyester chains, thereby improving hydrophilicity, increasing the number of available functional groups, and enhancing the comfort of treated fabrics. In the textile industry, esterases are used to modify the surface of polyester fabrics to improve absorbency, antistatic properties, dyeability, and comfort [7,8,34,35,36,37,38,39,40,41]. Lipases, a subgroup of serine hydrolases, hydrolyze the ester bonds of long-chain triglycerides into diglycerides, monoglycerides, fatty acids, and glycerol. They are highly stable and active in a wide range of organic solvents, which enables lipase-catalyzed reactions to occur under mild conditions such as low temperature, neutral pH, and atmospheric pressure [8]. Cutinases are serine esterases in the α/β hydrolase family, known for their ability to hydrolyze cutin, a natural plant cuticular polyester. They can hydrolyze the ester bonds of cutin, which is composed of hydroxy and epoxy fatty acids. It was later shown that they also catalyze the hydrolysis of various polymers, insoluble triglycerides, and soluble esters of low molecular weight [7,8,9,22,23,24,25,40].

Chitosan, a natural biopolymer derived from chitin, is composed mainly of 2-amino-2-deoxy-D-glucopyranose units linked by β-1,4 bonds. It is insoluble in water but dissolves in aqueous acidic solutions due to the dissociation of its amino groups. When alkali is added, it remains in solution up to a pH of 6.3, after which it forms a gel [42,43,44,45,46]. Its textile application, primarily due to its antimicrobial efficacy, depends on the origin of the chitin, the degree of deacetylation, the molecular weight distribution, the concentration and type of acid used, the pH, the ionic character, the temperature, and the available active groups on the textile material to which it is applied [46,47,48,49,50]. The antimicrobial efficacy of chitosan is based on the reaction of the NH_3_^+^ groups with the negatively charged cell membranes of microbes, and its efficacy is greater at low pH [48,49,50,51,52,53,54]. When applied to textiles, it is usually not resistant to textile care processes, so research on the durability of chitosan functionalization is ongoing. To improve the bonding of chitosan on textiles, various crosslinking agents are often used, which can enhance the stability of chitosan but may also affect the desired properties due to the reaction of the amino group with the crosslinking agent [46,55,56,57,58,59,60,61,62,63,64].

Knowledge of interfacial phenomena, especially zeta potential and surface charge, provides a fundamental theoretical basis for analyzing many important wet processes. For textile fibers in contact with aqueous electrolyte solutions, the electric double layer can form through dissociation of suitable molecular groups, preferential adsorption of certain ions, or both. Possible interactions—electrostatic, hydrophobic, Van der Waals, and London dispersion—between fibers and particles are mainly influenced by the surface charge of the fiber and the particle in solution. The electrokinetic double layer is characterized by the electrokinetic (zeta) potential of textile fibers. It plays an important role in the electrical characterization of textile materials, dyeing, and many other significant wet processes to which textile fibers are subjected [65,66,67]. Knowing the zeta potential and surface charge of fibers in textiles is important for monitoring or highlighting adsorption mechanisms, as these properties change with pretreatments and finishing processes [61,62,63,64,65,66,67,68,69,70,71]. For these reasons, this article investigated changes at the interface of cotton/polyester blended fabric with the attempt to bind chitosan without any crosslinking agent.

All textile finishing treatments must be environmentally friendly while maintaining the main properties of the textiles and must be durable for at least 3 to 50 washing cycles, depending on the intended use. Therefore, the bioactivation of cotton/polyester fabric with pectinase and/or esterase complex was performed and functionalized with homogenized chitosan solution prepared in 3% acetic acid, and its stability was investigated after three washing cycles in a nonionic surfactant. The changes were monitored by interfacial phenomena.

## 2. Materials and Methods

### 2.1. Materials

A 50/50 cotton/polyester blend fabric supplied by Čateks d.o.o. (Čakovec, Croatia) was used. The fabric was woven in a basket weave (Panama) 2/2, with a mass per unit area of 170 g/m^2^ and a fabric count of 38/22.

Acetic acid 99.5% p.a. (CH_3_COOH) and sodium hydroxide p.a. (NaOH) were purchased from Gram-mol d.o.o. (Zagreb, Croatia); potassium chloride p.a. (KCl) from Kemika (Zagreb, Croatia); cetylpyridinium chloride (CPC) and sodium dodecyl sulfate (SDS) from Sigma-Aldrich Co.—Merck KGaA (Darmstadt, Germany); and Felosan NOF (a fatty alcohol ethoxylates (FAEs) based nonionic surfactant, low-foaming stain remover and washing agent without APEO) from CHT/Bezema (Montlingen, Switzerland).

Beisol PRO, a specialized enzyme mixture from CHT/Bezema (Montlingen, Switzerland), was used for bioactivation of the cotton component in the blended fabric. This pectinase enzyme mixture operates optimally at neutral pH (pH 6–9) to remove starch size, enhance scouring, and prepare fabrics for dyeing. This product is bluesign^®^ approved and compliant with ZDHC MRSL v3.1 Level 3 standards.

Texazym PES (inoTEX, Dvur Kralove nad Labem, Czech Republic), a specialized enzymatic agent based on esterase, specifically containing ester hydrolase—lipolase with activity EC 3.1.1.3, was used for the bioactivation of the polyester component in blended fabric. This enzymatic agent modifies polyester fibers and enhances their hydrophilicity by forming new functional groups under mild processing conditions.

Oyster mushroom (*Pleurotus ostreatus*) chitosan, purchased from Chibio BioTECH (Qingdao, China) and donated by Tricomed SA (Lodz, Poland), was used in this research. It has a degree of deacetylation of 90%, a viscosity of 1000 cPs (mPa·s), and a molecular weight of 150 kDa.

### 2.2. Treatment Procedures

The pristine fabric was bioactivated in an enzymatic pretreatment using pectinase, esterase, and a mixture of both enzymes. Bioactivation was carried out with 2% owf (over weight of fabric) of enzyme (2% pectinase Beisol PRO, or 2% esterase Texazym PES, or 1% pectinase + 1% esterase) by the exhaustion method at 60 °C for 1 h in the drum of a machine Polycolor Turbomat P4502 (Mathis AG, Oberhasli, Switzerland), with an LR of 1:10 (liquor ratio).

Chitosan functionalization was performed according to [62]. A homogenized chitosan solution in 3% acetic acid was prepared as follows: 3 g/L of chitosan was added to a 3% acetic acid aqueous solution and stirred with a magnetic stirrer at room temperature for 24 h until a homogenized chitosan solution formed. For the functionalization of pristine and bioactivated cotton/polyester blended fabrics, the fabrics were impregnated (padded) in the homogenized chitosan solution with a wet pickup of 100%, dried at 110 °C for 2 min, and thermocondensed (cured) at 170 °C for 90 s. After functionalization, all fabrics were washed in distilled water and air-dried.

To determine the stability of chitosan functionalization, three washing cycles were performed in a Polycolor Turbomat P4502 machine with a liquor ratio of 1:10 at 60 °C for 30 min. The fabrics were washed with 1 g/L Felosan NOF to avoid interference with zeta potential measurements. After washing, the fabrics were rinsed and air-dried.

**Figure 1:**
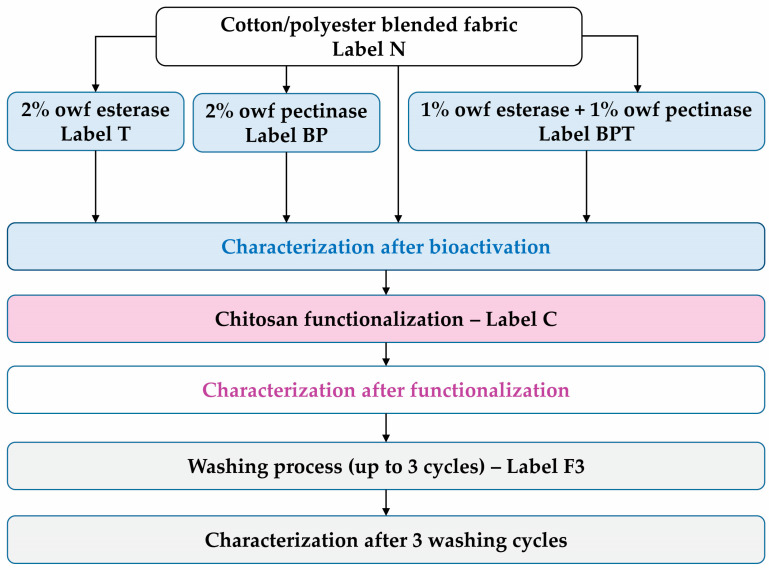
The process line diagram.

The schematic diagram of the process line is shown in Figure 1, indicating the appropriate labels of samples.

### 2.3. Characterization Methods

The mass per unit area (m) of the fabric in g/m^2^ was determined according to ISO 3801:1977 [72] using an analytical balance with an accuracy of 0.0001 g, model ALJ 220-5DNM (KERN & SOHN GmbH, Balingen, Germany). Sample size: 10 × 10 cm, 10 parallel samples. The change in mass per unit area (Δ*m*) was calculated relative to the pristine fabric.

Breaking force (F) in N and elongation at break (ε) in % were determined according to ISO 13934-1:2013 [73] on a Tensolab dynamometer (Mesdan S.p.A., Puegnago del Garda, Italy). Strip size: 50 mm × 200 mm, clamping distance 100 mm, testing speed of 100 mm/min, pretension 2 N, 5 parallel samples in the warp direction. Mechanical damage (U_m_) in % was calculated according to ISO 4312:1989 [74] using measured breaking force of pristine fabric and breaking force of fabrics after bio-activation/functionalization/washing process. The changes in elongation were calculated with respect to the pristine fabric.

The degree of whiteness (W_CIE_), in accordance with ISO 105-J02:1997 [75], was automatically calculated from the spectral remission, R [%], measured using a remission spectrophotometer Spectraflash SF300 (Datacolor AG, Lucerne, Switzerland). Measurements were performed under standard D65 light, with sphere geometry d/8°, using the “Measuring until tolerance” command in the Datacolor Tools program, version v1.3R4, which requires at least 10 measurements.

An independent samples *t*-test was conducted to evaluate differences in whiteness, spectral remission, mass per unit area, and breaking force, with the significance level set at α = 0.05. All statistical analyses were performed using TIBCO Statistica^®^ 14.1.0.

A USB digital microscope, IAN 490136_2501 (OWIM GmbH & Co., Neckarsulm, Germany), with a high-quality zoom function, was used to analyze the surface of blended fabrics. High-quality images with a resolution of 640 × 480 px were taken at 50× and 1000× magnification using HiView2.2 for Windows software.

The zeta potential (ZP, ζ, electrokinetic potential) in mV was calculated using the Helmholtz–Smoluchowski equation after measuring the streaming potential in an Adjustable Gap Cell of the SurPASS electrokinetic analyzer (Anton Paar GmbH, Graz, Austria) [65]. For each point, two parallel samples were measured four times at each pH value. A 0.001 mol/L KCl electrolyte solution was used for measurements in the pH range of 9 to 2, and the isoelectric point (IEP) was determined.

A specific surface charge (*q*) in mC was calculated using the back-titration method [65,69] with an automatic titration unit Titrino 736 (Metrohm AG, Herisau, Switzerland) and an ionic surfactant electrode 6.0507.120 (Metrohm AG, Herisau, Switzerland). CPC was used as the cationic polyelectrolyte, and SDS as the anionic polyelectrolyte solution. For the calculation, three fabric samples dwelt in the solution, and 9 titrations were performed.

Using the standard configuration of the goniometer Drop Shape Analyzer DSA30S from KRÜSS GmbH (Hamburg, Germany), the dynamic advancing contact angle with water (CA) in ° was determined. According to ISO 19403-6:2024 [76], a sample size of 4 × 4 cm, and three different drop measurements are required by the standard. During each measurement, the DSA30S dispenses a 10 µL drop and takes at least 20 photos over 2 s. Afterwards, the software determines the contact angle from each photo, calculates CA, and the standard deviation.

Wetting time (WT) in seconds and maximum wetted radius (MWR) in mm were determined according to AATCC TM 195-2017 [77] using the Moisture Management Tester (MMT) M290 (SDL Atlas, Rock Hill, SC, USA). Five parallel samples, each of size 8 × 8 cm, were tested. WT and MWR were calculated along with their coefficients of variation (CV), and the fabric type as identified by the MMT M290 software is provided.

The antimicrobial activity was determined against Gram-positive bacteria *Staphylococcus aureus* ATCC 6538 (*S. aureus*), Gram-negative bacteria *Escherichia coli* ATCC 8739 (*E. coli*), and microfungi *Candida albicans* ATCC 10231 (*C. albicans*) using ISO 20645:2004 [78] adapted accordingly. The test is based on measuring the inhibition zone around the sample and bacterial growth underneath it.

## 3. Results and Discussion

In this study, the interfacial phenomena of cotton/polyester blended fabric modified with enzymes and chitosan were investigated. As most research on surface modification of cotton/polyester blended fabrics has focused on polyester fibers, this study examined the influence of bioactivation on both fibers. Two commercially available enzyme complexes were used: a pectinase complex (Biosol PRO) for cotton, an esterase complex (Texazym PES) for polyester, and their mixture. This enzymatic pretreatment (bioactivation) aimed to activate the surface and improve interfacial properties, primarily the hydrophilicity of the polyester component in the blend. For the functionalization of bioactivated blended fabrics, a homogenized chitosan solution in a 3% acetic acid was applied using a pad–dry–cure process. The chitosan functionalization was performed by impregnation (pad–dry–cure technique) rather than as a surface coating, allowing the chitosan solution to penetrate the fiber structure while preserving the inherent flexibility and comfort (breathability) of the blended fabrics [79,80]. The effects were evaluated by electrokinetic analysis (zeta potential and isoelectric point), specific surface charge, contact angle, as well as wetting time and maximum wetted radius—measured using a Moisture Management Tester (MMT). Before analyzing interfacial phenomena, changes in the fabrics’ mechanical and spectral properties were determined using standard methods. Antimicrobial efficacy was tested as well.

### 3.1. Mechanical and Spectral Properties

**Figure 2:**
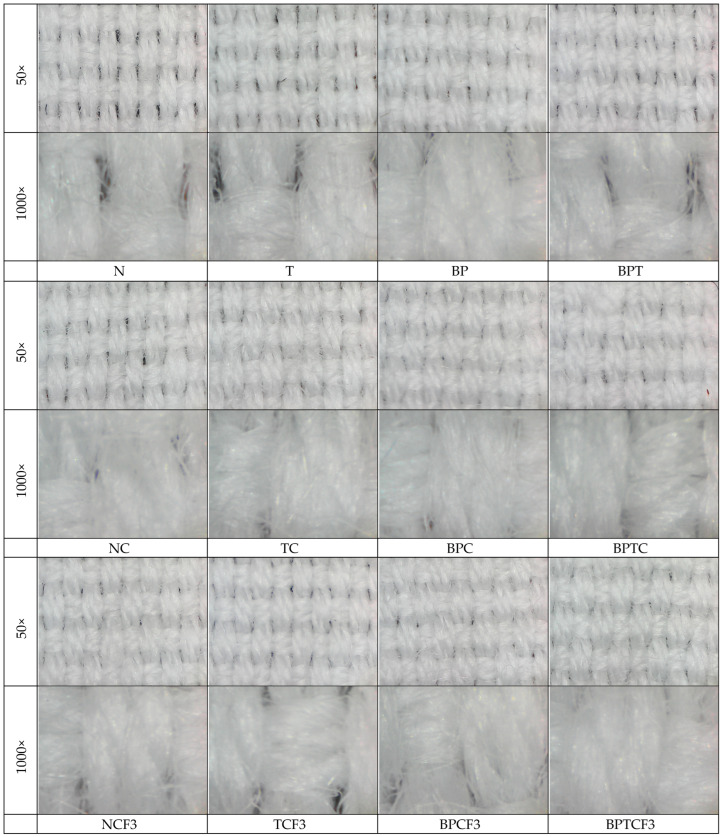
Digital microscope images of the cotton/polyester fabric surface before and after enzyme bioactivation, after chitosan functionalization, and after 3 washing cycles at 50× and 1000× magnification.

Results of tensile properties, including breaking force, elongation at break, mechanical damage, and mass per unit area, are presented in Table 1 and Table 2. Results for the fabrics’ spectral properties, including the maximum spectral remission at 700 nm and the degree of whiteness, are shown in Table 2. Digital microscope images at 50× and 1000× magnification of the surface of cotton/polyester fabrics before and after enzyme bioactivation, after chitosan functionalization, and after 3 washing cycles are shown in Figure 2.

From the results in Table 2, it can be seen that the stated mass per unit area of 170 g/m^2^ of pristine fabric was confirmed. Mass per unit area increased during enzymatic pretreatment, even though the fabric surface was modified. It is well known that cotton fibers are hydrophilic and, when exposed to heat and moisture, they swell and relax to their natural, denser state. During the washing process or wet treatment, heat, moisture, and mechanical agitation cause the fibers to tighten, resulting in fabric shrinkage after drying. This effect is more pronounced if the fabric is dried in a dryer [81,82,83,84]. Since only the cellulose component in the blend swells, the difference between the enzymes used, pectinase and/or esterase, can be observed. Esterase, which specifically targets polyester (Sample T), does not affect cellulose, resulting in a mass increase of less than 1%. In contrast, treatment with pectinase removes certain impurities, enabling greater fiber swelling and leading to a mass increase of 1.91%. When both enzymes are applied, the mass increase reaches 3.90%. This also suggests that, in addition to acting on polyester, esterase affects the cotton component, likely by degrading residual waxes and the cuticle layer on the cotton fibers [22,23,24,25]. Digital microscope images of the cotton/polyester fabric surfaces (Figure 2) further support these findings, showing that pectinase bioactivated fabrics (BP and BPT) have denser, thicker yarns, confirming that swelling occurred. An independent samples *t*-test revealed no statistically significant difference in mass per unit area values (*p* > 0.05). The slight increase in fabric mass per unit area after the combined treatments indicates an additive rather than a synergistic effect, as the observed change does not exceed the sum of the individual treatment contributions.

Polyester fibers are known for their excellent mechanical properties. The breaking force is highest for the pristine fabric (N) at 1096 N. When esterase is applied (Sample T), surface hydrolysis occurs, and the cleavage of polymer chains increases the number of terminal hydroxyl (–OH) and carboxyl (–COOH) groups. However, this chain shortening results in lower strength, and the mechanical damage is 7.3% for Sample T. Pectinase application results in mechanical damage 5.93% (Sample BP). The most favorable results were obtained using a combination of enzymes (1% pectinase + 1% esterase), where polyester hydrolysis occurred to a lesser extent than with esterase or pectinase alone. Additionally, the swelling and shrinkage of the cotton component contributed to maintaining fabric strength, resulting in only 2.55% mechanical damage for Sample BPT.

Functionalization with homogenized chitosan solution increased the mass per unit area by approximately 6%, as expected. At the same time, it slightly decreased the strength of the pristine fabric (1.73% for NC), while for bioactivated fabrics, it improved the strength. For enzyme bioactivated fabrics, the improvement was 5.6% for Sample TC, 6.6% for Sample BPC, and 4% for Sample BPTC. This confirms that homogenized chitosan reinforces the yarns in the fabric.

Elongation at break increases consistently following enzymatic bioactivation and chitosan functionalization, reflecting enhanced flexibility of the fabrics. For example, BPT exhibits a 25.54% increase in elongation, and BPC reaches 16.20% (+40.26%), indicating that fiber swelling and loosening of the yarn structure allow the fabric to stretch more before breaking.

In functionalized bioactivated cotton/polyester fabrics, chitosan binds to new active groups on the surface of the polyester fibers and forms a chitosan layer on the fabric surface, increasing the mass per unit area and reducing the strength loss caused by hydrolysis. During the washing process, the fabric is worn out, as seen in sample NF3 (*U_m_* = 7.66%), but because a neutral non-ionic surfactant (Felosan NOF) was used, shrinkage and mechanical damage are much less than when detergents are used. As a result, the strength loss after three washing cycles was only 1.55–5.75%. Partial removal of chitosan during washing was expected with a slight reduction in breaking force, yet values remain higher than the washed pristine fabric (NF3), indicating that chitosan persists on the fabric surface and that strong physical interactions between chitosan and fibers are maintained. This is consistent with the observed changes in mass per unit area: an additional ~2% increase was recorded for NCF3 and BPTCF3, as well as for the pristine NF3 fabric, whereas TCF3 and BPCF3 showed only a 0.5–0.6% increase, suggesting more limited chitosan deposition.

Elongation at break results further demonstrate that fabrics retain improved flexibility after three washing cycles. In particular, BPCF3 reaches an elongation of 21.15% (+83.12%), indicating that the enzyme combination in bioactivation and chitosan solution pad-dry cure treatment produces a stable functionalization that preserves the intrinsic textile properties, including tensile strength and flexibility, even after repeated washing. The increased elongation, along with low mechanical damage, highlights that these treatments enhance flexibility without compromising structural integrity, confirming the stability and effectiveness of the functionalization.

Similarly to mass per unit area, an independent samples *t*-test was conducted to examine differences in breaking force values. The results indicated no statistically significant difference (*p* > 0.05). These findings suggest that the combined effects are additive. Overall, these results indicate that combined enzyme bioactivation and chitosan functionalization exert an additive effect: they preserve breaking force, increase elongation, and maintain low mechanical damage, even after three washing cycles, demonstrating both the stability of functionalization and improved textile performance.

As treatment with acid can cause yellowing, the spectral remission was measured, and the degree of whiteness according to the International Commission on Illumination (fr. Commission Internationale de l’Eclairage, CIE) was calculated. From the results shown in Table 2, it can be seen that the untreated pristine cotton/polyester fabric has a degree of whiteness of 86.8. Enzymatic bioactivation has no significant influence on whiteness (W_CIE_ = 86.1–86.5). Functionalization with chitosan reduces the degree of whiteness due to the presence of yellowish chitosan. The pretreatment affects this outcome. When applied to the pristine fabric, the change in whiteness is small (W_CIE_ = 85.7), while for bioactivated fabric, it is W_CIE_ = 81.4–84.7. It should be noted that when esterase hydrolysis is performed, polyester is more activated, resulting in higher chitosan adsorption. Therefore, the whiteness is lowest, W_CIE_ = 81.4. During the washing process, up to three washing cycles, no change occurs, but there are slight changes due to mechanical movement. This is because, as already mentioned, a non-ionic surfactant was used instead of conventional detergents, so there was no influence on the degree of whiteness from oxidative bleaching agents or FWAs. The results of an independent samples *t*-test conducted to examine differences in whiteness and spectral remission values indicate that there was no statistically significant difference in whiteness and spectral remission values (*p* > 0.05).

### 3.2. Interfacial Phenomena

**Figure 3:**
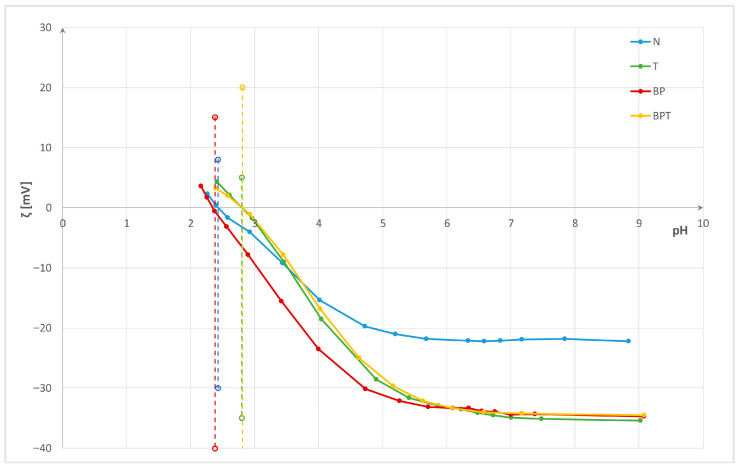
Zeta potential (ζ) of the pristine (N) and enzyme bioactivated cotton/polyester blended fabrics vs. pH of 0.001 M KCl. (dash lines mark IEPs).

**Figure 4:**
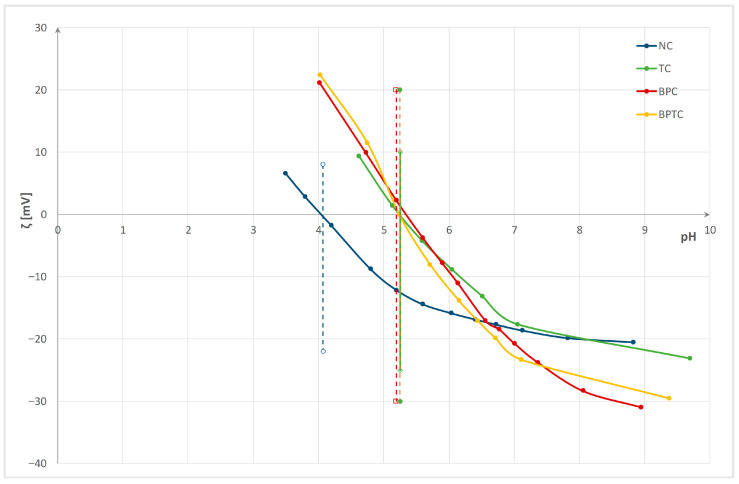
Zeta potential (ζ) of the pristine (N) and enzyme bioactivated cotton/polyester blended fabrics after functionalization vs. pH of 0.001 M KCl. (dash lines mark IEPs).

**Figure 5:**
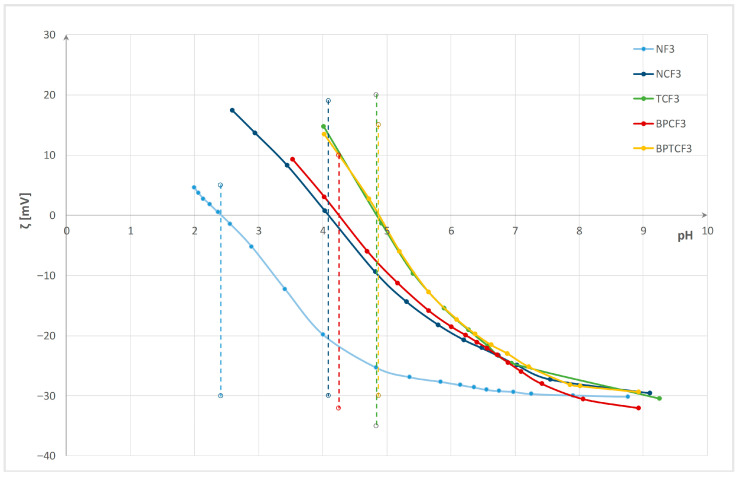
Zeta potential (ζ) of the pristine (N) and enzyme bioactivated cotton/polyester blended fabrics after functionalization and 3 washing cycles vs. pH of 0.001 M KCl. (dash lines mark IEPs).

Changes were monitored and evaluated by electrokinetic analysis (zeta potential and isoelectric point), specific surface charge, contact angle, as well as wetting time and maximum wetted radius measured using a MMT M290. Properties were evaluated after enzyme bioactivation, after chitosan functionalization, and after three washing cycles. The zeta potential (ζ) of the pristine fabric and enzyme-bioactivated cotton/polyester blended fabrics after functionalization and three washing cycles versus pH of 0.001 M KCl, with determined isoelectric points, are presented in Figure 3, Figure 4 and Figure 5. Interfacial phenomena—namely, isoelectric point (IEP), specific surface charge (q), water contact angle (CA), and its standard deviation for cotton/polyester fabrics before and after enzyme bioactivation, after chitosan functionalization, and after three washing cycles—are presented in Table 3. Wetting time, maximum wetted radius, and fabric type determined by the MMT M290 software are shown in Table 4.

The electrokinetic analysis was performed based on zeta potential (ζ, ZP) measurement on the electrokinetic analyzer SurPASS. From the results shown in Figure 3, it can be seen that pristine cotton/polyester fabric (N) has a zeta potential of ζ = −22.2 mV at a pH of 8.8, and IEP = 2.43. In neutral and alkaline solutions, cotton and polyester fibers in blends dissociate, resulting in a negative surface charge and negative zeta potential due to the presence of hydroxyl and carboxyl functional groups in cotton and carboxyl ester groups in polyester [65]. After the removal of non-cellulosic compounds (proteins, oils, waxes, pectin, carbohydrates, inorganic materials, etc.) during the scouring process, the hydroxyl groups of cotton are revealed. Additionally, chemical bleaching processes cause the formation of new surface groups (–CO, –CHO, and –COOH) in cotton [17,68,69,70,71,79,84]. Due to the high crystallinity of the fibers, the number of carboxyl groups in polyester fibers is very low, making the fiber surface hydrophobic and unable to absorb water molecules [7,40,65]. According to the literature, cotton shows an isoelectric point (IEP) at a pH lower than 2, while polyester has an IEP at a pH of 4 [62,63,64,65]. Blending fibers in the fabric causes a change, and the IEP is at pH 2.43. The surface properties of both fibers influence the zeta potential and surface charge. The specific surface charge (*q*) measured at neutral pH 7, as shown in Table 2, confirms a negative surface charge of −0.917 mC. Bioactivation decreases the zeta potential to −34.4 mV for pectinase and to −35.4 mV for esterase. Bioactivation with esterase creates new groups on the polyester component and cleans the surface of cotton, resulting in greater fabric swelling and shifting the IEP to pH 2.8. For that reason, the surface charge is less negative.

From the results in Figure 4, it can be seen that chitosan functionalization increases the zeta potential in alkaline and neutral electrolyte solutions and shifts the isoelectric point to a higher pH. The fabric surface becomes more positive, indicating a decrease in negative active groups, and the positive charge of chitosan arises from the dissociation of –NH_3_ groups [57,58,61,62,63]. For the functionalized pristine fabric (sample NC), ζ = −20.6 mV at pH 8.8, and the IEP is 4.07. In bioactivated fabrics, this effect is especially pronounced, indicating greater chitosan bonding. Considering the zeta potential at the pH plateau (pH 8–10), differences between enzyme pretreatments of chitosan-functionalized blended fabrics are evident. The chitosan-functionalized esterase bioactivated blended fabric (sample TC) has the highest zeta potential of −21.3 mV at pH 8.9, followed by the fabric pretreated with both enzymes (BPTC) at −28.0 mV at pH 9, and the fabric pretreated only with pectinase (BPC) at −30.9 mV at pH 8.9. The IEP changes accordingly: for TC and BPTC, the IEP is 5.25, and for BPC, the IEP is 5.19. The results for specific surface charge confirm these findings.

**Figure 6:**
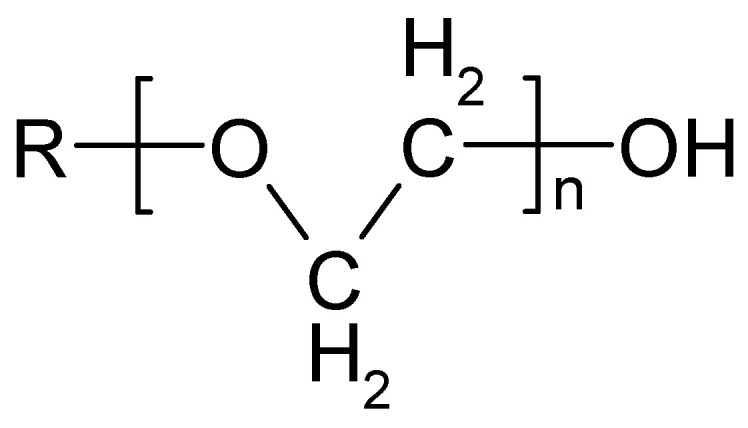
Structure of fatty alcohol ethoxylates (FAEs).

The washing process depends on chemical action, mechanical agitation, temperature, time, and water, which combines these factors [79,80,81]. In this study, all factors were kept the same and were carefully selected to ensure an appropriate evaluation of bioactivation and chitosan functionalization. The temperature was set to the maximum recommended for washing polyester, at 60 °C; the time was set to 30 min; the pH was adjusted to 7 (neutral); and soft water was used, as water hardness also has an influence. Instead of a conventional detergent, the non-ionic surfactant Felosan NOF was chosen. This specially developed auxiliary, based on fatty alcohol ethoxylate (Figure 6), offers excellent emulsifying and washing properties. Previous studies have confirmed weak hydrophobic interactions between non-ionic surfactants and chitosan in water [63,85]. All washing process factors were consistent, and the non-ionic surfactant used does not interact with chitosan. Therefore, the observed differences in interfacial phenomena can be attributed to the bioactivation, chitosan, and the cotton or polyester component of the blended fabric.

From the results in Figure 5, it can be seen that after three washing cycles, washed pristine cotton/polyester fabric (NF3) exhibits a more negative zeta potential (ZP) in neutral and alkaline media (−30 mV at pH 7–9) compared to the pristine fabric (N). This indicates that washing with Felosan NOF had a cleaning effect, although the ZP values remain higher than those of bioactivated fabrics. However, the isoelectric point (IEP) remains unchanged at 2.41. The washed fabric functionalized with chitosan shows a lower zeta potential at pH 7–9 than after functionalization. Although some chitosan was removed from the fabric surface, the zeta potentials of all chitosan-functionalized blended fabrics remain higher than before functionalization, indicating the presence of chitosan after three washing cycles. The IEP also confirms the presence of chitosan.

There is a difference in stability related to pretreatment among bioactivated fabrics. For the chitosan-functionalized pristine fabric after washing (NCF3), some chitosan was removed, exposing negative groups and resulting in a more negative ZP of −29.5 mV at pH 9, but the IEP remains at 4.09. The chitosan-functionalized esterase bioactivated fabric initially had the highest ZP, suggesting the highest chitosan content, but three washing cycles led to partial chitosan removal, causing a leftward shift in the IEP to 4.84. The zeta potential of the chitosan-functionalized pectinase bioactivated blended fabric did not change significantly in alkaline medium, but some changes occurred, and the IEP shifted leftward to 4.25 for BPCF3 and to 4.87 for BPTCF3.

It should be noted that esterase bioactivation (T and BPT) leads to better functionalization and greater stability. This is likely due to enzymatic hydrolysis of the polyester component, resulting in better wash resistance compared to the pristine fabric and the fabric bioactivated only with pectinase, as indicated by the shift in IEPs to higher pH values. This suggests that chitosan remains on the surface after three washing cycles, which is consistent with the mass per unit area results in Table 2. These findings are confirmed by the specific surface charges, which are all positive after three washing cycles.

The results of the interfacial phenomena presented in Table 3 indicate that pristine and enzyme bioactivated fabrics (N, T, BP, BPT) exhibit negative surface charge values (*q* = −0.660 to −0.917 mC) and low contact angles (20–41°), indicating hydrophilic behavior. Enzymatic bioactivation further enhances wettability, as reflected by the decrease in contact angle, with BPT showing complete wetting (CA ≈ 0°). The relatively high standard deviation values (e.g., BP: 25.89°) suggest rapid water absorption and non-uniform droplet spreading, making precise CA determination difficult. Textiles are heterogeneous and porous, and unlike films and foils, it is not always possible to determine the contact angle [70,71,84,86,87,88]. For heterogeneous and hydrophilic textiles, the wicking test method is more suitable [87].

Chitosan functionalized fabrics (NC, TC, BPC, BPTC) display a shift toward positive surface charge (q = 0.853–1.606 mC) and higher contact angles (83–126°), confirming successful chitosan deposition and increased surface hydrophobicity. This is consistent with the increase in isoelectric point (IEP), indicating modification of the surface chemistry. Lower standard deviation values for some samples (e.g., BPC: 4.72°) suggest more stable droplet formation and more uniform surface properties. The increase in apparent hydrophobicity after chitosan functionalization, despite the more positive surface charge, can be attributed to partial surface coverage and restructuring on the textile surface, including pore accessibility. As can be seen from Figure 2, pores are less accessible. The initial arrangement of chitosan chains and possible residual acetic acid interactions may temporarily reduce wettability as well. Importantly, after washing, hydrophilicity is restored, suggesting rearrangement or partial redistribution of the chitosan within the textile structure. After three washing cycles, all samples exhibit contact angles of 0°, indicating extremely rapid water uptake. This suggests that the fabric structure dominates the wetting behavior, and water droplets are immediately absorbed into the porous textile, preventing reliable CA measurement. Despite this, the IEP and surface charge values remain altered compared to pristine fabric, indicating that chitosan is still partially retained on the fiber surface.

Digital microscope images of the cotton/polyester fabric surfaces shown in Figure 2 clearly reveal changes in yarn morphology, including slight swelling and a denser, thicker appearance of the yarns, while the overall fabric porosity is preserved. The retention of porosity is further supported by moisture management measurements, which demonstrate that the treated fabrics maintains its breathability. At the same time, the observed yarn swelling is consistent with internal uptake of the chitosan solution rather than the formation of a surface film. The fabric also remains flexible after treatment, indicating that no rigid coating layer is formed and that the modification occurs within the fiber assembly.

The moisture management properties of heterogeneous, porous materials such as textiles depend on their water resistance, water repellency, water absorption, the wicking of fibers and yarns, and the geometric and internal structures of the constituent materials, such as fibers and yarns [86,87,88,89,90,91,92]. The Moisture Management Tester (MMT) is an instrument that objectively characterizes the wetting, wicking, spreading, and transfer of liquid moisture on and between fabric surfaces. It consists of upper and lower concentric moisture sensors that detect and measure changes in electrical resistance related to the water content in the fabric [91]. Since not all fabrics can form a contact angle (CA), the wetting time (WT), maximum wetted radius (MWR), and MMT type of cotton/polyester fabrics before and after enzyme bioactivation, after chitosan functionalization, and after three washing cycles were determined on the MMT. Results are collected in Table 4.

The moisture management properties of the cotton/polyester blend fabric presented in Table 4 as wetting time (WT), maximum wetted radius (MWR), and fabric type reveal a strong influence of enzyme bioactivation and chitosan functionalization on liquid transport behavior. The wetting time (WT) measured on the MMT is the period during which the upper and lower surfaces of the fabric just begin to get wet, corresponding to the drop absorption time in the drop test method. MWR is the maximum wetted radius at 2 min [88,92]. Results show that the pristine cotton/polyester blend fabric exhibits a moderate wetting time of 5.4 s, and a moisture spreading MWR of 23.25 mm, characteristic of moisture management fabrics, indicating good hydrophilicity.

Enzyme bioactivation reduces the wetting time to less than 2.5 s and increases MWR to 25 mm, indicating enhanced hydrophilicity and improved liquid transport. For enzyme bioactivated fabrics, the shorter wetting time results from surface changes on the fibers. Esterases perform surface hydrolysis, and the cleavage of polymer chains increases the number of terminal hydroxyl (–OH) and carboxyl (–COOH) groups in the polyester. Additionally, some esterases can hydrolyze the waxes on cotton fibers. This change enables better absorption in the polyester component, as water binds to the new chemical groups formed during pretreatment. Fabric type by MMT is given based on measured moisture management properties considering the wetting, adsorption, and maximum wetted radius on the bottom side, and the transport through the fabric. The pristine cotton/polyester fabric type is “Moisture Management Fabric,” which confirms its suitability for applications that require effective moisture management. Pectinase bioactivation does not change the fabric type, but it improves hydrophilicity. However, esterase pretreatment of the polyester component results in fast wetting, absorption, and spreading, achieving a maximum wetting radius of 25 mm. Therefore, blends treated with this enzyme (samples T and BPT) are characterized as “Fast Absorbing and Quick Drying Fabric”.

In contrast, chitosan-functionalized fabrics (NC, TC, BPC, BPTC) show slower wetting and reduced spreading, particularly NC (WT_MMT_ = 14.75 s; MWR = 7.50 mm), which is classified as a “Water Penetration Fabric,” meaning it does not allow water to penetrate through the fabric but does allow evaporation (fabric breathing). This behavior suggests that chitosan forms a surface layer that limits immediate liquid spreading and alters capillary transport within the fabric structure. The functionalized fabric bioactivated with both enzymes (BPTC) also shows a slower WT of 12.801 s, but the MWR is 11.25 mm, so it transports moisture and is classified as a “Moisture Management Fabric.” The other two functionalized bioactivated fabrics have faster WTs and an MWR of 21.25 mm, so both are also classified as “Moisture Management Fabrics.” It can be said that combined enzymatic bioactivation and chitosan functionalization partially restore moisture management behavior, indicating a balance between surface modification and internal wettability.

After three washing cycles, all fabrics exhibit improved performance, with low wetting times (≈2.39–3.29 s) and high spreading radii (up to 25.00 mm). Most fabrics are classified as “Fast-Absorbing and Quick-Drying Fabrics”, demonstrating that washing enhances liquid transport, likely due to partial removal or redistribution of chitosan and increased accessibility of hydrophilic sites. An additional reason can be the use of nonionic FAE surfactants in washing. It can be assumed that the hydrophobic tail (fatty alcohol) of the FAE surfactant interacts with the hydrophobic surface of polyester and chitosan, while the hydrophilic ethoxylate (polyoxyethylene) chain extends into the aqueous phase [93]. As with any surfactant, this improves wettability and reduces surface tension. Therefore, after 3 washing cycles, the fabrics do not form a contact angle.

Overall, the results indicate that enzymatic bioactivation enhances wettability, hydrophilicity, and moisture transport, while chitosan functionalization modifies surface charge and initially reduces liquid spreading by promoting absorption; however, after washing, the porous textile structure dominates liquid behavior, resulting in rapid wetting and effective moisture management, thereby confirming the stability of the functionalization.

### 3.3. The Antimicrobial Efficacy

The antimicrobial efficacy of blended fabrics against *S. aureus* (ATCC 6538), *E. coli* (ATCC 8739), and *C. albicans* (ATCC 10231) was evaluated according to ISO 20645:2004. In accordance with the standard, antimicrobial efficacy is determined by observing the presence of an inhibition zone and/or the absence of microbial growth beneath and on the surface of the sample. The results are presented in Table 5 using this evaluation criterion: “+” indicates visible antimicrobial activity (presence of an inhibition zone), “+/−” indicates antimicrobial activity without an inhibition zone but with no microbial growth beneath the sample, and “−” indicates no antimicrobial activity.

The pristine fabric exhibited no antimicrobial activity against the tested microorganisms, as expected [59,60,61,62,63,64,94]. However, after 3 washing cycles, antimicrobial activity against *S. aureus* and *E. coli* was observed, suggesting that the washing agent, nonionic surfactant Felosan NOF, contributed to antibacterial efficacy. Nonionic surfactants exhibit limited direct antimicrobial activity compared to cationic surfactants; however, they can act as antimicrobial enhancers by increasing the permeability of bacterial cell membranes and facilitating lipid emulsification. They reduce microbial adhesion to surfaces, thereby contributing to a protective coating effect. Their mode of action involves interaction with proteins and phospholipid membranes, leading to increased membrane permeability and leakage of low-molecular-weight compounds. This leakage may result in cell damage or death due to the loss of essential ions and amino acids [95,96].

According to the applied evaluation criteria, enzyme bioactivated fabrics (T, BP, BPT) exhibit limited activity, with T and BPT showing “+/−” against *S. aureus*, indicating inhibition of growth beneath the sample without the formation of a visible inhibition zone, while no activity (−) is observed against *E. coli* and *C. albicans*. The reason may lie in the esterase used for fabric bioactivation. Esterases and related lipases can hydrolyze insoluble long-chain triglycerides or fats and therefore have the ability to hydrolyze membrane lipids, which increases bacterial cell membrane permeability and leads to cell death [97,98,99].

As chitosan is a non-leaching antimicrobial agent, its efficacy is primarily evaluated based on the inhibition of microbial growth in direct contact with the fabrics. All chitosan-functionalized fabrics (NC, TC, BPC, BPTC) demonstrate antimicrobial activity (“+/−”) against all tested microorganisms, confirming effective inhibition of microbial growth in the contact area, although without visible inhibition zones. This behavior is consistent with the mechanism of chitosan, which acts through contact-based interactions with microbial cell walls rather than by diffusion into the surrounding medium [59,60,61,62,63,64,94].

After three washing cycles, antimicrobial activity is retained in all chitosan-functionalized fabrics (NCF3, TCF3, BPCF3, BPTCF3), as indicated by “+/−” results for all microorganisms, demonstrating good stability of treatment.

Overall, the results indicate that chitosan treatment provides stable antimicrobial efficacy, primarily through contact inhibition, while enzymatic bioactivation mainly facilitates surface activation and has a limited direct antimicrobial effect.

### 3.4. The Interactions Between Chitosan and Cotton/Polyester Blended Fabric

Based on the results, knowledge from previous authors’ research, and existing literature, the interactions between chitosan and cotton/polyester blended fabric can be explained by intermolecular forces, surface chemistry, and fiber morphology. Chitosan is a cationic polysaccharide that becomes positively charged in acidic environments due to the protonation of its amino groups (–NH_2_ → –NH_3_^+^). This cationic nature enables strong interactions with negatively charged functional groups on cotton and/or polyester through several mechanisms.

**Figure 7:**
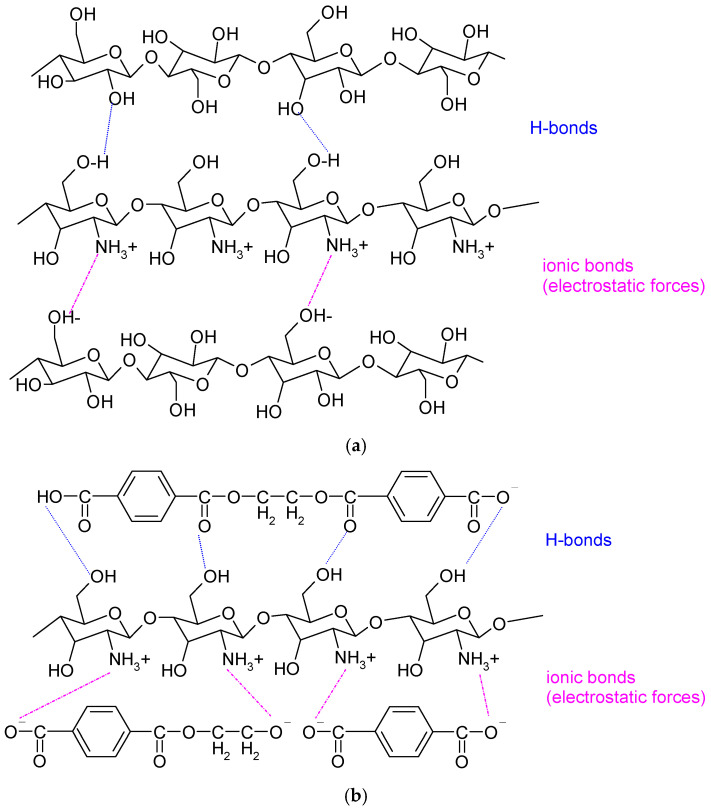
Chitosan bonding to (**a**) cotton component, and (**b**) polyester [63] component of blended fabric.

Cotton, primarily composed of cellulose, has a high number of hydroxyl (–OH) groups available on the fiber surface. These groups can participate in extensive hydrogen bonding, particularly N–H···O interactions between the protonated amino groups of chitosan and the hydroxyl groups of cellulose. These hydrogen bonds are the dominant force governing compatibility and stability in cellulose–chitosan systems. Additionally, depending on environmental conditions, cellulose surfaces may exhibit a slight negative charge, allowing electrostatic (ionic) attractions with chitosan’s –NH_3_^+^ groups (Figure 7a). Physical entanglement of chitosan chains within the porous fiber network further contributes to adhesion and retention, reinforcing the overall interaction.

For polyester (represented in Figure 7b by the most common polyethylene terephthalate, PET), the surface is initially hydrophobic and chemically inert, which limits interaction with chitosan. However, enzymatic treatments—particularly with esterases—can induce mild surface hydrolysis, generating polar functional groups such as carboxyl (–COOH/–COO^−^) and hydroxyl (–OH). This modification increases wettability and introduces negatively charged sites. As a result, electrostatic interactions can occur between the positively charged chitosan (–NH_3_^+^) and negatively charged polyester surface groups (–COO^−^), significantly enhancing chitosan bonding to the fiber.

In both systems, the combination of hydrogen bonding and ionic (electrostatic) interactions acts as a form of physical crosslinking. These non-covalent interactions collectively form a three-dimensional network that distributes binding throughout the material rather than localizing it at the surface. This distributed interaction network enhances the mechanical stability, durability, and functional performance of the chitosan-treated cotton/polyester blended fabric. In cellulose-based systems, hydrogen bonding is dominant, while in modified polyester systems, ionic interactions become more prominent due to surface functionalization.

Overall, hydrogen bonding, electrostatic interactions, and physical entanglement explain the strong affinity of chitosan for both cotton and enzyme-modified polyester fibers, forming the basis for its effective application in textile functionalization.

## 4. Conclusions

The interfacial and functional properties of cotton/polyester blended fabric modified by enzyme bioactivation and chitosan functionalization were systematically investigated. Enzymatic bioactivation with pectinase complex (Biosol PRO), and/or esterase complex (Texazym PES), individually or combined, resulted in fabric surface modification without compromising intrinsic fiber strength and improved flexibility, as the enzymes cannot penetrate the fiber due to their size; thus, enzymatic hydrolysis is limited to the fiber surface. Meanwhile, chitosan functionalization altered surface charge, improved tensile properties, and enhanced antimicrobial efficacy. Mechanical damage during washing occurred due to abrasion, wear, and partial removal of chitosan, but the achieved tensile strength values remain higher than those of washed pristine cotton/polyester fabric.

Hydrogen bonding, electrostatic interactions, and physical entanglement account for the strong affinity of chitosan for both cotton and enzyme-modified polyester fibers, forming the basis for its effective use in textile functionalization. The treatment was applied by impregnation (pad–dry–cure), enabling modification within the fiber structure rather than forming a surface coating, thus preserving fabric flexibility and handle. As a result, interfacial effects are indirectly reflected in macroscopic mechanical properties, where improved tensile strength and elongation indicate enhanced fiber–fiber interactions and a more resilient internal structure.

Enzymatic bioactivation did not change the degree of whiteness, but increased hydrophilicity and moisture transport, while chitosan initially reduced the degree of whiteness and liquid spreading but enabled controlled absorption. After washing, the porous fabric structure governed liquid behavior, resulting in rapid wetting and effective moisture management, confirming the washing stability of the chitosan functionalization.

The combined treatments—enzymatic bioactivation and chitosan functionalization—exhibited an additive effect, maintaining strength, enhancing elongation, and limiting mechanical damage even after repeated washing. The best performance was achieved with chitosan functionalization on esterase-bioactivated fabrics. Since the fabrics retained antimicrobial activity after washing, they are suitable for use in hospital environments, such as cloths for covering instruments or protective overcoats. The pad–dry–cure technique is particularly suitable since it preserves flexibility, softness, and air permeability. The retention of fabric porosity and confirmed breathability, as demonstrated through moisture management testing, indicate that the treated materials remain appropriate for real-world applications, such as functional textiles, where both comfort and performance are essential. Furthermore, enzyme-based bioactivation provides an environmentally friendly and industrially feasible alternative to conventional alkaline treatments (cotton scouring and polyester hydrolysis), allowing straightforward integration into existing textile processing equipment.

## Figures and Tables

**Table 1:** Tensile properties: breaking force (*F*), mechanical damage (*U_m_*), and elongation at break (ε) of cotton/polyester fabrics before and after enzyme bioactivation, after chitosan functionalization, and after 3 washing cycles.

Fabric	*F* [N]	CV [%]	*U_m_* [%]	*U_m_* _pairs_ [%]	*ε* [%]	CV [%]	Δ*ε* [%]	Δ*ε*_pairs_ [%]
N	1096.0	2.53	0.00		11.55	1.837	0.00	
T	1016.0	1.23	7.30		12.60	3.367	9.09	
BP	1031.0	2.02	5.93		14.25	1.489	23.38	
BPT	1068.0	0.39	2.55		14.50	1.439	25.54	
NC	1077.0	3.61	1.73		15.45	1.373	33.77	
TC	1089.0	1.66	0.64	−7.19	14.85	4.285	28.57	17.86
BPC	1104.0	1.01	−0.73	−7.08	16.20	5.238	40.26	13.68
BPTC	1110.0	0.00	−1.28	−3.93	15.15	4.201	31.17	4.48
NF3	1012.0	2.06	7.66		13.93	1.692	20.63	
NCF3	1079.0	4.31	1.55	−0.19	19.33	3.170	67.39	
TCF3	1048.0	0.86	4.38	3.76	18.90	0.000	63.64	27.27
BPCF3	1067.0	2.14	2.65	3.35	21.15	5.015	83.12	30.56
BPTCF3	1033.0	5.84	5.75	6.94	17.70	11.985	53.25	16.83

**Table 2:** Mass per unit area (*m*) and its changes (Δ*m*), maximum spectral remission at 700 nm (R_max_), and the degree of whiteness (W_CIE_) of cotton/polyester fabrics before and after enzyme bioactivation, after chitosan functionalization, and after 3 washing cycles.

Fabric	*m* [g/m^2^]	Δ*m* [%]	Δ*m*_pairs_ [%]	R_max_ at 700 nm [%]	W_CIE_
N	169.81	0.00		89.71	86.8
T	171.38	0.92		87.68	86.1
BP	173.06	1.91		88.00	86.3
BPT	176.43	3.90		88.22	86.5
NC	179.96	5.98		88.27	85.7
TC	180.67	6.40	5.42	88.14	81.4
BPC	181.74	7.03	5.02	87.83	84.7
BPTC	179.45	5.68	1.71	88.55	84.1
NF3	172.96	1.86		88.33	86.5
NCF3	184.92	8.90	2.76	87.35	85.5
TCF3	181.37	6.81	0.39	87.40	83.0
BPCF3	182.78	7.64	0.57	87.73	85.1
BPTCF3	183.29	7.94	2.14	87.92	84.6

**Table 3:** Interfacial phenomena: isoelectric point (IEP), specific surface charge (*q*), contact angle (CA), and its standard deviation of cotton/polyester fabrics before and after enzyme bioactivation, after chitosan functionalization, and after 3 washing cycles.

Fabric	*IEP*	*q* [mC]	CA [°]	StDev CA [°]
N	2.43	−0.917	41.28	22.44
T	2.80	−0.766	20.04	17.53
BP	2.38	−0.660	24.04	25.89
BPT	2.81	−0.718	0.00	0.00
NC	4.07	0.853	126.26	7.89
TC	5.25	1.606	83.26	16.96
BPC	5.19	1.202	125.31	4.72
BPTC	5.25	1.361	107.77	23.92
NF3	2.41	−0.831	0.00	0.00
NCF3	4.09	0.728	0.00	0.00
TCF3	4.84	1.424	0.00	0.00
BPCF3	4.25	1.019	0.00	0.00
BPTCF3	4.87	0.954	0.00	0.00

**Table 4:** The moisture management properties: Wetting time (WT), maximum wetted radius (MWR), and MMT type of cotton/polyester fabrics before and after enzyme bioactivation, after chitosan functionalization, and after 3 washing cycles.

Fabric	WT_MMT_ [s]	CV	MWR [mm]	CV	MMT Type
N	5.351	0.544	23.75	0.105	Moisture Management Fabric
T	2.390	0.068	25.00	0	Fast-Absorbing and Quick-Drying Fabric
BP	2.437	0.063	25.00	0	Moisture Management Fabric
BPT	2.461	0.078	25.00	0	Fast-Absorbing and Quick-Drying Fabric
NC	14.750	0.576	7.50	1.150	Water Penetration Fabric
TC	10.762	0.255	21.25	0.120	Moisture Management Fabric
BPC	4.246	0.484	21.25	0.210	Moisture Management Fabric
BPTC	12.801	0.105	11.25	0.220	Moisture Management Fabric
NF3	2.391	0.104	25.00	0	Moisture Management Fabric
NCF3	2.648	0.053	25.00	0	Fast-Absorbing and Quick-Drying Fabric
TCF3	2.547	0.023	25.00	0	Fast-Absorbing and Quick-Drying Fabric
BPCF3	2.582	0.023	25.00	0	Fast-Absorbing and Quick-Drying Fabric
BPTCF3	3.289	1.890	18.75	0.667	Fast-Absorbing and Quick-Drying Fabric

**Table 5:** The antimicrobial activity of fabrics after bioactivation, chitosan functionalization, and 3 washing cycles.

Fabric	*S. aureus*	*E. coli*	*C. albicans*
N	−	−	−
T	+/−	−	−
BP	−	−	−
BPT	+/−	−	−
NC	+/−	+/−	+/−
TC	+/−	+/−	+/−
BPC	+/−	+/−	+/−
BPTC	+/−	+/−	+/−
NF3	+/−	+/−	−
NCF3	+/−	+/−	+/−
TCF3	+/−	+/−	+/−
BPCF3	+/−	+/−	+/−
BPTCF3	+/−	+/−	+/−

+ antimicrobial activity (inhibition zone can be observed); +/− antimicrobial activity (no colonies beneath); − no antimicrobial activity.

## Data Availability

The raw data supporting the conclusions of this article will be made available by the authors on request.

## References

[1] Gulzar T., Farooq T., Kiran S., Ahmad I., Hameed A., Shahidul I., Butola B.S. (2019). 1—Green chemistry in the wet processing of textiles. The Impact and Prospects of Green Chemistry for Textile Technology.

[2] Sheikh J., Bramhecha I., Shahidul I., Butola B.S. (2019). 6—Enzymes for green chemical processing of cotton. The Impact and Prospects of Green Chemistry for Textile Technology.

[3] Araújo R., Casal M., Cavaco-Paulo A. (2008). Application of enzymes for textile fibres processing. Biocatal. Biotransform..

[4] Kumar D., Bhardwaj R., Jassal S., Goyal T., Khullar A., Gupta N. (2023). Application of enzymes for an eco-friendly approach to textile processing. Environ. Sci. Pollut. Res..

[5] Mamun Kabir S., Koh J., Ferreira Mendes K., Nogueira de Sousa R., Cabral Mielke K. (2022). Sustainable Textile Processing by Enzyme Applications. Biodegradation Technology of Organic and Inorganic Pollutants.

[6] Quartinello F., Guebitz G.M., Ribitsch D. (2019). Surface functionalization of polyester. Meth. Enzymol..

[7] Čorak I., Pušić T., Tarbuk A. (2019). Enzimi za hidrolizu poliestera. Tekstil.

[8] Houde A., Kademi A., Leblanc D. (2004). Lipases and Their Industrial Applications: An Overview. Appl. Biochem. Biotechnol..

[9] Chen S., Su L., Chen J., Wu J. (2013). Cutinase: Characteristics, preparation, and application. Biotechnol. Adv..

[10] Guebitz G.M., Cavaco-Paulo A. (2008). Enzymes go big: Surface hydrolysis and functionalization of synthetic polymers. Trends Biotechnol..

[11] Bristi U., Pias A., Lavlu F.H. (2019). A sustainable process by bio-scouring for cotton knitted fabric suitable for next generation. J. Text. Eng. Fash. Technol..

[12] Niaz A., Malik Q.J., Muhammad S., Shamim T., Asghar S. (2011). Bioscouring of cellulosic textiles. Color. Technol..

[13] Hardin I.R., Li Y. (1997). Enzymatic Scouring of Cotton: Effects of Structure and Properties. Text. Chem. Color..

[14] Grancarić A.M., Pušić T., Lesić-Domšić B., Plantić L. (2001). The Impact of Treating Cotton with Alkali Pectinases on Cotton Knitted Fabric Sewability. Tekstil.

[15] Jordanov I., Mangovska B. (2001). Optimal Parameters of Enzymatic Scouring and Compared with Alkaline Scouring. Tekstil.

[16] Zulić D., Grancarić A.M. (2002). Alkali Pectinases Used in Cotton Scouring. Tekstil.

[17] Forte-Tavčer P., Preša P. (2004). Pretreatment of Cotton with Pectinases and Peracetic Acid. Tekstil.

[18] Grancarić A.M., Pušić T., Tarbuk A. (2006). Enzymatic Scouring for Better Textile Properties of Knitted Cotton Fabrics. J. Nat. Fibers.

[19] Kalantzi S., Mamma D., Christakopoulos P., Kekos D. (2008). Effect of pectate lyase bioscouring on physical, chemical and low-stress mechanical properties of cotton fabrics. Bioresour. Technol..

[20] Pušić T., Tarbuk A., Dekanić T. (2015). Bio-innovation in cotton fabric scouring—Acid and neutral pectinases. Fibres Text. East. Eur..

[21] Guo Z., Zhou Y., Wang P., Wang Q., Xu B., Zhou M., Cui L., Zhang L., Yu Y. (2026). An innovative method for the removal of impurities and bleaching of cotton fabrics by UV/H_2_O_2_/pectinase: Intrinsic mechanisms and treatment effects. Int. J. Biol. Macromol..

[22] Degani O., Gepstein S., Dosoretz C.G. (2002). Potential use of cutinase in enzymatic scouring of cotton fiber cuticle. Biochem. Biotechnol..

[23] Agrawal P.B., Nierstrasz V.A., Bouwhuis G.H., Warmoeskerken M.M.C.G. (2008). Cutinase and pectinase in cotton bioscouring: An innovative and fast bioscouring process. Biocatal. Biotransform..

[24] Agrawal P.B., Nierstrasz V.A., Warmoeskerken M.M.C.G. (2008). Role of mechanical action in low-temperature cotton scouring with *F. solani pisi* cutinase and pectate lyase. Enzym. Microb. Technol..

[25] Degani O. (2021). Synergism between Cutinase and Pectinase in the Hydrolysis of Cotton Fibers’ Cuticle. Catalysts.

[26] Hsieh Y.L., Pastore C., Kiekens P. (2001). Surface Characteristics of Polyester Fibers. Surface Characteristics of Fibers and Textiles.

[27] Gawish S.M., Bourgeois M., Ambroise G. (1986). Effect of Cationic Surfactants on the Alkaline Hydrolysis of Polyester Fabrics. Am. Dyest. Report..

[28] Grancarić A.M., Soljačić I., Rukavina I., Čavar T. (1988). Influence of the Procedure of Treatment on the Effects of Alkaline Hydrolysis of Polyester. Tekstil.

[29] Zeronian S.H., Collins M.J. (1989). Surface Modification of Polyester by Alkaline Treatments. Text. Prog..

[30] Kallay N., Grancarić A.M., Tomić M. (1990). Kinetics of Polyester Fibre Dissolution. Text. Res. J..

[31] Grancarić A.M., Kallay N. (1993). Kinetics of polyester fiber alkaline hydrolysis: Effect of temperature and cationic surfactants. J. Appl. Polym. Sci..

[32] Čorak I., Tarbuk A., Đorđević D., Višić K., Botteri L. (2022). Sustainable Alkaline Hydrolysis of Polyester Fabric at Low Temperature. Materials.

[33] Dong Z.Q., Chen G.Q. (2012). Alkaline Hydrolysis of Polyester in the Presence of Ionic Liquids. Adv. Mater. Res..

[34] Tkavc T., Vesel A., Acero E.H., Fras Zemljič L. (2012). Comparison of oxygen plasma and cutinase effect on polyethylene terephthalate surface. J. Appl. Polym. Sci..

[35] Alisch M., Feuerhack A., Müller H., Mensak B., Andreaus J., Zimmermann W. (2004). Biocatalytic modification of polyethylene terephthalate fibres by esterases from actinomycete isolates. Biocatal. Biotransform..

[36] Wu J., Cai G., Liu J., Ge H., Wang J. (2014). Eco-friendly surface modification on polyester fabrics by esterase treatment. Appl. Surf. Sci..

[37] Kardas I., Lipp.-Symonowicz B., Sztajnowski S., Wojciechowska D. (2014). The influence of PET fibres surface enzymatic modification on the selected properties. Autex Res. J..

[38] Donelli I., Taddei P., Smet P.F., Poelman D., Nierstrasz V.A., Freddi G. (2009). Enzymatic surface modification and functionalization of PET: A water contact angle, FTIR, and fluorescence spectroscopy study. Biotechnol. Bioeng..

[39] Abo El-Ola S.M., Moharam M.E., El-Bendary M.A. (2013). Optimum conditions for surface modification of PET by lipase enzymes produced by *Egyptian bacilli* in comparison with standard one. Indian J. Fibre Text. Res..

[40] Eberl A., Heumann S., Brückner T., Araujo R., Cavaco-Paulo A., Kaufmann F., Kroutil W., Guebitz G.M. (2009). Enzymatic surface hydrolysis of poly(ethylene terephthalate) and bis(benzoyloxyethyl) terephthalate by lipase and cutinase in the presence of surface active molecules. J. Biotechnol..

[41] Frasoński T., Tarbuk A., Matyjas-Zgondek E. (2025). Bio-innovative functionalization of knitted polyester fabrics using enzyme, TiO_2_ and fluorescent whitening agents for sustainable architectural textiles. Holist. Approach Environ..

[42] Shabbir M., Rather L.J., Mohammad F., Ahmed S., Ikram S. (2017). Chitosan: Sustainable and Environmental-Friendly Resource for Textile Industry. Chitosan: Derivatives, Composites and Applications.

[43] El Knidri H., Belaabed R., Addaou A., Laajeb A., Lahsini A. (2018). Extraction, chemical modification and characterization of chitin and chitosan. Int. J. Biol. Macromol..

[44] Furuike T., Komoto D., Hashimoto H., Tamura H. (2017). Preparation of chitosan hydrogel and its solubility in organic acids. Int. J. Biol. Macromol..

[45] Sikorski D., Gzyra-Jagiela K., Draczynski Z. (2021). The Kinetics of Chitosan Degradation in Organic Acid Solutions. Mar. Drugs.

[46] Hahn T., Bossog L., Hager T., Wunderlich W., Breier R., Stegmaier T., Zibek S., van den Broek L.A.M., Boeriu C.G. (2019). Chitosan Application in Textile Processing and Fabric Coating. Chitin and Chitosan: Properties and Applications.

[47] Bakshi P.S., Selvakumar D., Kadirvelu K., Kumar N.S. (2020). Chitosan as an environment friendly biomaterial—A review on recent modifications and applications. Int. J. Biol. Macromol..

[48] Islam S.U., Butola B.S. (2019). Recent advances in chitosan polysaccharide and its derivatives in antimicrobial modification of textile materials. Int. J. Biol. Macromol..

[49] Latańska I., Kolesińska B., Draczyński Z., Sujka W. (2020). The use of chitin and chitosan in manufacturing dressing materials. Prog. Chem. Appl. Chitin Its Deriv..

[50] Sikorski D., Bauer M., Frączyk J., Draczyński Z. (2022). Antibacterial and Antifungal Properties of Modified Chitosan Nonwovens. Polymers.

[51] Zhang Z., Chen L., Ji J., Huang Y., Chen D. (2003). Antibacterial Properties of Cotton Fabrics Treated with Chitosan. Text. Res. J..

[52] Ke C.L., Deng F.S., Chuang C.Y., Lin C.H. (2021). Antimicrobial actions and applications of chitosan. Polymers.

[53] Rabea E.I., Badawy M.E.T., Stevens C.V., Smagghe G., Steurbaut W. (2003). Chitosan as antimicrobial agent: Applications and mode of action. Biomacromolecules.

[54] Verlee A., Mincke S., Stevens C.V. (2017). Recent development in antibacterial and antifungal chitosan and its derivatives. Carbohydr. Polym..

[55] Hsieh S.H., Chen W.H., Wei L.L. (2003). A spectroscopic analysis of the reaction mechanism of polycarboxylic acids’ crosslinking with chitosan and cotton fabric. Cellul. Chem. Technol..

[56] Chung Y.-S., Lee K.-K., Kim J.-W. (1998). Durable Press and Antimicrobial Finishing of Cotton Fabrics with a Citric Acid and Chitosan Treatment. Text. Res. J..

[57] Sunder E., Nalankilli G. (2014). Croslinking of Chitosan with Cotton using Polycarboxylic Acids. Int. J. Eng. Res. Technol..

[58] Draczyński Z., Flinčec Grgac S., Dekanić T., Tarbuk A., Boguń M. (2017). Implementation of Chitosan into Cotton Fabric. Tekstilec.

[59] Flinčec Grgac S., Tarbuk A., Dekanić T., Sujka W., Draczyński Z. (2020). The Chitosan Implementation into Cotton and Polyester/Cotton Blend Fabrics. Materials.

[60] Flinčec Grgac S., Biruš T.-D., Tarbuk A., Dekanić T., Palčić A. (2023). The Durable Chitosan Functionalization of Cellulosic Fabrics. Polymers.

[61] Tarbuk A., Flinčec Grgac S., Dekanić T., Čorak I., Begović S. (2023). The Influence of Cotton/Polyester Blend Fabric Pre-treatment on Chitosan Functionalization. Eng. Power.

[62] Čorak I., Tarbuk A., Flinčec Grgac S., Dekanić T. (2024). Bio-Innovative Modification of Poly(Ethylene Terephthalate) Fabric Using Enzymes and Chitosan. Polymers.

[63] Čorak I., Tarbuk A., Dekanić T., Sikorski D., Draczyński Z. (2024). The Functionalization of Activated Polyester Fabrics with Chitosan—Changes in Zeta Potential and Moisture Management. Materials.

[64] Pušić T., Kaurin T., Liplin M., Budimir A., Čurlin M., Grgić K., Sutlović A., Volmajer Valh J. (2023). The Stability of the Chitosan Coating on Polyester Fabric in the Washing Process. Tekstilec.

[65] Grancarić A.M., Tarbuk A., Pušić T. (2005). Electrokinetic properties of textile fabrics. Color. Technol..

[66] Luxbacher T., Pušić T., Bukšek H., Petrinić I. (2016). The zeta potential of textile fabrics: A review. Tekstil.

[67] Luxbacher T., Kozłowski R.M., Mackiewicz-Talarczyk M. (2020). Electrokinetic Properties of Natural Fibres. Handbook of Natural Fibres.

[68] Stana-Kleinschek K., Strnad S., Ribitsch V. (1999). Surface characterization and adsorption abilities of cellulose fibers. Polym. Eng. Sci..

[69] Tarbuk A., Grancarić A.M., Leskovac M. (2014). Novel cotton cellulose by cationization during mercerization—Part 2: The interface phenomena. Cellulose.

[70] Grancarić A.M., Tarbuk A., Hadžić S., Simončič B. (2023). From Raw to Finished Cotton—Characterization by Interface Phenomena. AUTEX Res. J..

[71] Peršin Z., Stana-Kleinschek K., Sfiligoj-Smoje M., Kreže T., Ribitsch V. (2004). Determining the Surface Free Energy of Cellulose Materials with the Power Contact Angle Measurement. Text. Res. J..

[72] (1977). Textiles—Woven fabrics—Determination of Mass per Unit Length and Mass per Unit Area.

[73] (2013). Textiles—Tensile Properties of Fabrics—Part 1: Determination of Maximum Force and Elongation at Maximum Force Using the Strip Method.

[74] (1989). Surface Active Agents—Evaluation of Certain Effects of Laundering—Methods of Analysis and Test for Unsoiled Cotton Control Cloth.

[75] (1997). Textiles—Tests for Colour Fastness—Part J02: Instrumental Assessment of Relative Whiteness.

[76] (2024). Paints and Varnishes—Wettability—Part 6: Measurement of Dynamic Advancing and Receding Angle by Changing the Volume of a Drop.

[77] (2017). Liquid Moisture Management Properties of Textile Fabrics.

[78] (2004). Textile Fabrics—Determination of Antibacterial Activity—Agar Diffusion Plate Test.

[79] Omerogullari Basyigit Z. (2021). Application Technologies for Functional Finishing of Textile Materials. Textiles for Functional Applications.

[80] Schindler W.D., Hauser P.J., Schindler W.D., Hauser P.J. (2004). 2—Chemical finishing processes. Woodhead Publishing Series in Textiles, Chemical Finishing of Textiles.

[81] Jakobi G. (1987). Detergents and Textile Washing.

[82] Smulders E. (2002). Laundry Detergents.

[83] Soljačić I., Pušić T. (2005). Textile Care, Book 1: Water Medium Cleaning.

[84] Čorak I., Tarbuk A., Marković J., Grgić K. (2021). Changes in sorption properties due to multiple washing processes. Tekstil.

[85] Pepić I., Filipović-Grčić J., Jalšenjak I. (2009). Bulk properties of nonionic surfactant and chitosan mixtures. Colloids Surf. A Physicochem. Eng. Asp..

[86] El Mogahzy Y.E. (2009). 11—Finishing processes for fibrous assemblies in textile product design. Woodhead Publishing Series in Textiles, Engineering Textiles.

[87] Tarbuk A., Flinčec Grgac S., Dekanić T. (2019). Wetting and wicking of hospital protective textiles. Adv. Technol..

[88] Tegegne W., Haile A. (2025). Improving hydrophilicity and comfort characteristics of polyester/cotton blend fabric through lipase enzyme treatment. Clean Technol. Environ. Policy.

[89] Tarbuk A., Begović S., Guxho G., Spahiu T.K., Xhafka E., Gjeta A., Sulejmani A. (2026). Comfort Properties of Enzymatically Pretreated Cotton-Polyester Blended Fabrics. Proceedings of the Joint International Conference: 5th Conference on Engineering and Entrepreneurship and 11th Textile Conference, ITC-ICEE 2025, Tirana, Albania, 23–24 October 2025.

[90] Šaravanja A., Dekanić T., Pušić T., Volmajer Valh J. (2023). The effect of accelerated ageing simulation on the properties of polyester fabrics. Tekstil.

[91] SDL Atlas—MMT^®^: (Moisture Management Tester), M290. Brochure. https://sdlatlas.com/products/mmt-moisture-management-tester.

[92] Yao B., Li Y., Hu J., Kwok Y., Yeung K. (2006). An improved test method for characterizing the dynamic liquid moisture transfer in porous polymeric materials. Polym. Test..

[93] Romero-Cano M.S., Martín-Rodríguez A., de las Nieves F.J. (2000). Adsorption and Desorption of Triton X-100 in Polystyrene Particles with Different Functionality: II. Desorption Study. J. Colloid Interface Sci..

[94] Rahman Bhuiyan M.A., Hossain M.A., Zakaria M., Islam M.N., Zulhash Uddin M. (2017). Chitosan Coated Cotton Fiber: Physical and Antimicrobial Properties for Apparel Use. J. Polym. Environ..

[95] Tobe S., Majima T., Tadenuma H., Suekuni T., Sakai K., Sakai H., Abe M. (2015). Nonionic Surfactants Enhancing Bactericidal Activity at Their Critical Micelle Concentrations. J. Oleo Sci..

[96] Adewuyi A., Ayodele Oderinde R., Ololade Ademisoye A. (2013). Antibacterial Activities of Nonionic and Anionic Surfactants From the Seed Oil of Citrullus lanatus. Jundishapur J. Microbiol..

[97] Hetrick K.J., Raines R.T. (2022). Assessing and utilizing esterase specificity in antimicrobial prodrug development. Methods Enzymol..

[98] Hetrick K.J., Aguilar Ramos M.A., Raines R.T. (2021). Endogenous Enzymes Enable Antimicrobial Activity. ACS Chem. Biol..

[99] Prabhawathi V., Boobalan T., Sivakumar P.M., Doble M. (2014). Antibiofilm Properties of Interfacially Active Lipase Immobilized Porous Polycaprolactam Prepared by LB Technique. PLoS ONE.

